# Do Differences in Levels, Types, and Duration of Muscle Contraction Have an Effect on the Degree of Post-exercise Depression?

**DOI:** 10.3389/fnhum.2016.00159

**Published:** 2016-04-29

**Authors:** Shota Miyaguchi, Sho Kojima, Hikari Kirimoto, Hiroyuki Tamaki, Hideaki Onishi

**Affiliations:** Institute for Human Movement and Medical Sciences, Niigata University of Health and WelfareNiigata, Japan

**Keywords:** post-exercise depression, muscle contraction levels, muscle contraction types, movement duration, transcranial magnetic stimulation

## Abstract

We conducted two experiments to determine how differences in muscle contraction levels, muscle contraction types, and movement duration affect degree of post-exercise depression (PED) after non-exhaustive, repetitive finger movement. Twelve healthy participants performed repetitive abduction movements of the right index finger at 2 Hz. In experiment 1, we examined the effects of muscle contraction levels at 10, 20, and 30% maximum voluntary contraction and the effects of muscle contraction types at isotonic and isometric contraction. In experiment 2, we examined the effects of movement duration at 2 and 6 min. Motor-evoked potentials (MEPs) were recorded from the right first dorsal interosseous muscle before movement tasks and 1–10 min after movement tasks. MEP amplitudes after isotonic contraction tasks were significantly smaller than those after isometric contraction tasks and decreased with increasing contraction levels, but were independent of movement duration. This study demonstrated that the degree of PED after non-exhaustive repetitive finger movement depended on muscle contraction levels and types. Thus, the degree of PED may depend on the levels of activity in the motor cortex during a movement task. This knowledge will aid in the design of rehabilitation protocols.

## Introduction

In the field of rehabilitation, repetitive motor tasks by voluntary or passive movement are widely used to enhance muscle strength, to improve range of motion, and to promote motor learning or motor function in patients after stroke. Brasil-Neto et al. (1993) were the first to report a decrease in primary motor cortex excitability after exhaustive exercise that causes muscle fatigue, with other studies reporting a similar phenomenon (Liepert et al., 1996; Samii et al., 1996; Sacco et al., 2000; Zijdewind et al., 2000; Lentz and Nielsen, 2002). Brasil-Neto et al. (1993) referred to this phenomenon as post-exercise depression (PED). A number of studies reported that PED persists for 20–30 min after exhaustive exercise by maximum voluntary contraction (MVC) (McKay et al., 1995; Zanette et al., 1995; Liepert et al., 1996). Because spinal reflexes and peripheral and subcortical components [i.e., H wave, F wave, M wave, and motor evoked potential (MEP) by transcranial electrical stimulation] do not change, PED may be caused by intracortical mechanisms (Brasil-Neto et al., 1993; Samii et al., 1996; Baumer et al., 2002; Bridoux et al., 2015). Potential mechanisms for PED include long-term depression, decreased neurotransmitter levels, decreased excitability of intracortical glutamatergic networks, and increased excitability of inhibitory GABAergic networks (Zanette et al., 1995; Samii et al., 1996; Teo et al., 2012b).

However, some studies reported that PED is also induced after non-exhaustive movements for a short time, as well as after exhaustive movement, which causes muscle fatigue. Recently, we determined that PED is induced after index finger abduction movement of 10% MVC at 0.5 Hz for 10 min (Miyaguchi et al., 2013). Moreover, Teo et al. (2012b) observed PED after 10 s of non-exhaustive finger flexion-extension movement that persisted for 8 min after the movement. This observation made it clear that PED is induced after non-exhaustive movement as well as after exhaustive movement. Contrary to this observation, Samii et al. (1997) found that PED was induced after exhaustive movement but not after non-exhaustive movement. However, a few recent studies agreed with the findings of earlier studies that reported that PED is also induced after non- exhaustive movement (Teo et al., 2012b; Miyaguchi et al., 2013). Although Zanette et al. (1995) demonstrated that PED is induced only in the area of active muscles in the primary motor cortex, other studies reported that PED is induced in not only the area of active muscles in primary motor cortex but also in the area of inactive adjacent muscles (Bonato et al., 1996; Teo et al., 2012b). In addition, while Bonato et al. (1996) and Humphry et al. (2004) reported that PED is induced not only in the area of exercised muscles but also in the area of contralateral non-exercising homonymous muscles, Baumer et al. (2002) reported that PED is not induced in the area of contralateral non-exercising homonymous muscle. Moreover, although Samii et al. (1996) demonstrated that PED persists for only 4 min after a movement task resulting in fatigue, other studies reported that PED persists for 30 min after a movement task not resulting in fatigue (Zanette et al., 1995; Bonato et al., 2002). As these studies indicate, many discrepancies exist in previous studies on PED.

We speculate that the degree of PED that persists after a movement task may be associated with differences in movement tasks and target muscles. Previous studies have already indicated that the degree of PED that persists after a movement task is associated with movement frequency and movement limb (i.e., the dominant or non-dominant hand; Teo et al., 2012a,b). However, whether differences in muscle contraction levels, muscle contraction types, and duration of movement affect the degree of PED has not yet been clarified. In sum, investigation of movement tasks that affect the degree of PED is important because it elucidates how the mechanism of PED works.

The purpose of this study was to determine whether differences in levels, types, and duration of muscle contraction affect the degree of PED persistence in movement tasks. We suggested that the degree of PED persistence is a factor in the levels of activity in the motor cortex during movement tasks (Miyaguchi et al., 2013). Accordingly, we hypothesized that the degree of PED persistence increases with increasing the activity in the motor cortex during movement tasks. This study is clinically relevant because it helps elucidate the effect of different parameters of muscle contractions on PED, providing clinicians with better fundamental knowledge when designing rehabilitation protocols.

## Experimental Procedure

### Participants

Twelve healthy subjects [*n* = 10 men; *n* = 2 women; age 23.3 ± 2.7 (mean ± standard deviation); range, 21–29 years] participated in this study. All subjects provided written, informed consent. The study was approved by the ethics committee at the Niigata University of Health and Welfare and conformed to Declaration of Helsinki guidelines. During the two experiments, subjects were comfortably seated with the right shoulder in slight abduction, elbow in 90° flexion, and forearm in a pronated position.

### TMS and MEP Recording

Motor-evoked potentials were used to evaluate corticomotor excitability before and after repetitive, non-exhaustive finger movement. A Magstim 200 (Magstim Co, Dyfed, UK) was used as a magnetic stimulator, and a figure eight TMS coil (diameter, 9.5 cm) was placed tangentially at approximate 45° from the midline with the handle facing posterolaterally on each subject’s skull.

The optimal position for eliciting MEPs from FDI was carefully determined by delivering Visor 2 TMS Neuro-navigation (EEMAGINE Medical Imaging Solutions GmbH, BER, DE), which could correctly identify the position of the primary motor cortex by monitoring each subject’s fMRI image. The intensity of the stimulator output was adjusted for baseline recording so that the average stimulus produced an MEP of 1 mV in the relaxed FDI muscle in at least 50% of 10 stimuli. TMS was delivered at 0.25 Hz in 20 trials before motor tasks (pre) and in 15 trials to 1 min from 10 min after motor tasks (post).

### EMG Recording

Surface EMG was recorded from the right FDI using Ag/AgCl electrodes. The recording and reference electrodes were placed over the muscle and tendon, respectively. The signals were amplified (×100) by a pre-amplification system (A-DL-720-140, 4 Assist, Tokyo, Japan) and digitized at 10 kHz by an A/D converter (PowerLab 8/30, AD Instruments, Colorado Springs, Colorado, USA). The EMG signals were filtered with a 15-Hz high-pass filter and rectified and smoothed during motor tasks. Data were recorded and stored for off-line analysis (Lab Chart 7, AD Instruments) on a personal computer.

### Movement Tasks

#### Experiment 1: Effects in Muscle Contraction Levels and Types

In experiment 1, we investigated whether PED was affected by differences in muscle contraction levels and types in motor tasks. All 12 subjects participated in this experiment. The subjects performed repetitive abduction movements of the right index finger for 2 min. The movements were applied at 2 Hz using auditory feedback. The muscle contraction levels were set at 10, 20, and 30% MVC. The muscle contraction types were set at either isotonic contraction or isometric contraction. The six motor tasks were set as follows: (1–3) repetitive isotonic contraction at 10, 20, and 30% MVC (isotonic_10, isotonic_20, and isotonic_30% MVC, respectively), and (4–6) repetitive isometric contraction at 10, 20, and 30% MVC (isometric_10, isometric _20, and isometric_30% MVC, respectively). Under the isotonic condition, the subjects performed repetitive abduction movements of the right index finger, and movement ranged from the neutral position to the end range of abduction. The weights as 10, 20, and 30% MVC were loaded during motor tasks (Miyaguchi et al., 2013). The weights were adjusted to make the demanded contraction levels equal to the average amplitude of the smoothing EMG signals during the motor tasks. Under the isometric condition, subjects were limited to movement of the metacarpophalangeal joint by affixing the right index finger to the laboratory table and repetitively contracting the right FDI following auditory feedback at 2 Hz. Prior to each movement task, subjects practiced repetitive abduction movements by watching EMG signals on a personal computer (PC) screen for approximately 10 s to perform appropriate motor tasks. Then, the subjects performed repetitive abduction movements without watching EMG signals on a PC screen during motor tasks. MEPs before movement tasks (pre) were started to take measurements approximately 10 min after motor practice. The six motor tasks were randomly performed in a repeated measures design, with a break of at least 10 min between each condition. The subjects began to perform the next motor task after MEP amplitudes returned to pre-exercise values, thereby excluding the influence of the preceding motor task.

#### Experiment 2: Effect in Movement Duration

In experiment 2, we investigated whether PED was affected by differences in the movement duration of motor tasks. Ten of the 12 subjects [*n* = 9 men; *n* = 1 women; age 23.8 ± 3.0 (mean ± standard deviation); range, 21–29 years] participated in this experiment. Subjects performed repetitive abduction movements of the right index finger at 2 Hz in the same fashion as in experiment 1. Movement durations were set at 2 and 6 min, respectively. The contraction level was 10% MVC and contractions were isotonic. Because finger movement at 2 Hz for 6 min with 20 or 30% MVC induce muscle fatigue, we decided to perform motor task only with 10% MVC. The two motor tasks were randomly performed in a repeated measures design, with a break of at least 1 week between each condition.

### Data and Statistical Analyses

Motor-evoked potentials amplitudes, except the maximum and minimum MEP amplitudes, were calculated from peak-to-peak amplitudes of 20 trials (pre) and 15 trials (post). The MEP ratio (the MEP amplitudes after movement task/the MEP amplitudes at pre × 100) was calculated to evaluate the change in degree of PED. The average amplitudes of the smoothing EMG signals for 60 s after the beginning of motor tasks (start_60 s) and for 60 s before the end of motor tasks (end_60 s) were normalized with the values obtained at MVC in each subject. In experiment 1, a one-way repeated analysis of variance (ANOVA) was used to compare the MEP amplitudes in pre. A three-way repeated ANOVA was used to compare MEP ratio (factor 1 = CONTRACTION LEVEL, factor 2 = CONTRACTION TYPE, factor 3 = TIME). Moreover, if CONTRACTION LEVEL × TIME interaction, the CONTRACTION TYPE × TIME interaction and/or CONTRACTION LEVEL × CONTRACTION TYPE × TIME interaction were observed, two-way repeated measures ANOVA was used to compare MEP ratio. In experiment 2, a two-way repeated ANOVA was used to compare the change in MEP amplitudes (factor 1 = MOVEMENT DURATION, factor 2 = TIME). *Post hoc* analysis was performed by Fisher’s LSD method. A paired *t*-test was used to compare the MEP amplitudes in pre, and average amplitudes of smoothing EMG signals at 2 and 6-min task. In both experiments, a paired *t*-test was used to compare the average amplitudes of the smoothing EMG signals at start_60 s and end_60 s. Differences were considered statistically significant at *P* < 0.05 for all analyses.

## Results

### Experiment 1: Effects in Muscle Contraction Levels and Types

**FIGURE 1:**
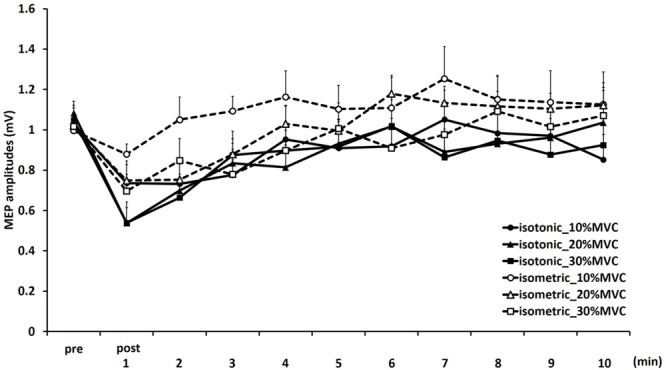
**Means and standard errors of the motor-evoked potentials (MEP) amplitudes before movement tasks (pre) and 1–10 min after movement tasks at each task in experiment 1.** Continuous lines indicate isotonic contraction tasks; dotted lines indicate isometric contraction tasks. Circles, triangles, and squares indicate 10, 20, and 30% MVC tasks, respectively.

**FIGURE 2:**
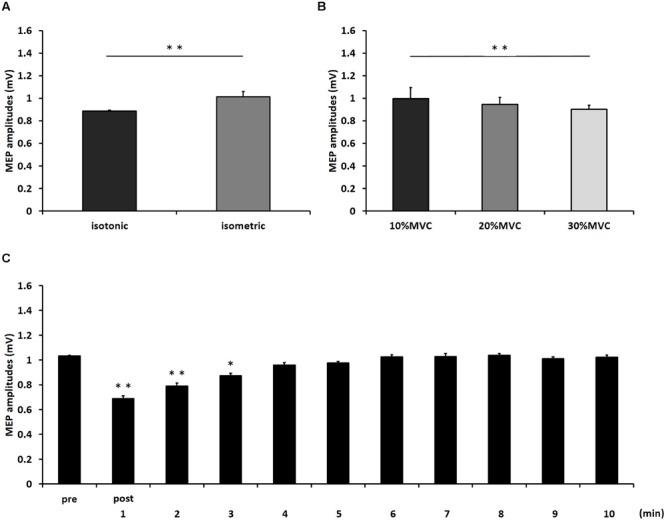
**The results of *post hoc* analyses in three-way repeated measures ANOVA used to compare MEP amplitudes from pre to post-10 min. (A)** Comparison of means and standard errors of the MEP amplitudes at differences in contraction types (^∗∗^*P* < 0.01). **(B)** Comparison of means and standard errors of the MEP amplitudes at differences in contraction levels (^∗∗^*P* < 0.01). **(C)** Comparison of means and standard errors of the MEP amplitudes at different times after movement tasks [^∗^*P* < 0.05, ^∗∗^*P* < 0.01 (vs. pre)].

**FIGURE 3:**
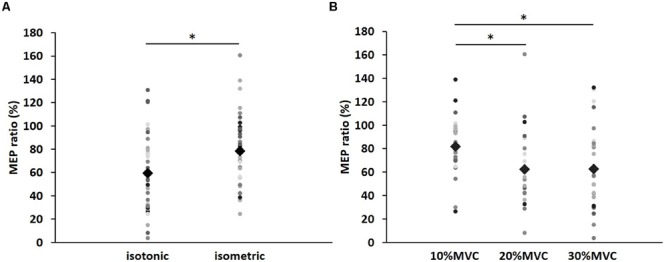
**The results of *post hoc* analyses in two-way repeated measures ANOVA used to compare MEP ratio at post-1 min. (A)** Comparison of means and standard errors of the MEP ratio at differences in contraction types (^∗^*P* < 0.05). **(B)** Comparison of means and standard errors of the MEP ratio at differences in contraction levels (^∗^*P* < 0.05). Circle indicates the MEP ratio of all subjects. Rhombus indicates mean MEP ratio.

**Figure 1** shows the mean MEP amplitudes induced by TMS under all conditions. No statistical difference was found among the MEP amplitudes at pre [*F*_(5,55)_ = 0.307, *P* = 0.907]. A three-way repeated measures ANOVA revealed significant main effects of CONTRACTION LEVEL [*F*_(2,715)_ = 4.420, *P* < 0.05]; CONTRACTION TYPE [*F*_(1,715)_ = 24.046, *P* < 0.01] (**Figure 2A**); and TIME [*F*_(10,715)_ = 7.169, *P* < 0.01]. No significant differences were found between the CONTRACTION LEVEL × CONTRACTION TYPE interaction [*F*_(2,715)_ = 2.010, *P* = 0.135]; the CONTRACTION LEVEL × TIME interaction [*F*_(20,715)_ = 0.525, *P* = 0.957]; the CONTRACTION TYPE × TIME interaction [*F*_(10,715)_ = 0.598, *P* = 0.817]; and the CONTRACTION LEVEL × CONTRACTION TYPE × TIME interaction [*F*_(20,715)_ = 0.350, *P* = 0.997]. *Post hoc* analyses demonstrated that the MEP amplitudes at the 30% MVC condition were significantly smaller than those at the 10% MVC condition (*P* < 0.01; **Figure 2B**). No significant differences were found between the 10% MVC condition and 20% MVC condition (*P* = 0.103) and between the 20% MVC condition and 30% MVC condition (*P* = 0.181). Moreover, the MEP amplitudes at post-1 min (*P* < 0.01), post-2 min (*P* < 0.01), and post-3 min (*P* < 0.05) were significantly smaller than those at pre (**Figure 2C**). Because time factor had many levels in this study and significant differences in MEP amplitudes were observed between pre and post-3 min, we performed three-way repeated measures ANOVA to compare MEP ratio at post-1, post-2, and post-3 min. **Table 1** shows the average MEP ratio at post-1, post-2, and post-3 min, and the results of three-way and two-way repeated measures ANOVA. The results of three-way repeated measures ANOVA revealed significant main effects of the CONTRACTION LEVEL factor, the CONTRACTION TYPE factor, the TIME factor and statistical significance between the CONTRACTION LEVEL × CONTRACTION TYPE × TIME interaction but not the other interactions. Therefore, we attempted a two-way repeated ANOVA to compare the MEP ratio at post-1, post-2, and post-3 min. The results revealed significant main effects of the CONTRACTION LEVEL factor and the CONTRACTION TYPE factor but not the CONTRACTION LEVEL × CONTRACTION TYPE interaction at post-1 min. **Figure 3** shows the results of *post hoc* analyses. The results demonstrated that the MEP ratio at the isotonic contraction condition were significantly smaller than those at the isometric contraction condition (*P* < 0.05), and the MEP ratio at the 20% MVC condition and 30% MVC condition were significantly smaller than those at the 10% MVC condition (*P* < 0.05). A two-way repeated measures ANOVA revealed significant main effects of the CONTRACTION TYPE factor but not the CONTRACTION LEVEL factor and the CONTRACTION LEVEL × CONTRACTION TYPE interaction at post-2 min, and *post hoc* analyses demonstrated that the MEP ratio at the isotonic condition were significantly smaller than those at the isometric condition (*P* < 0.05). At post-3 min, a two-way repeated measures ANOVA revealed no significant main effects (CONTRACTION TYPE factor, CONTRACTION LEVEL factor) and interaction.

**Table 1:** Motor-evoked potentials (MEP) ratio (mean ± standard error) from post-1 min to post-3 min, the results of a three-way repeated measures ANOVA from post-1 min to post-3 min, and a two-way repeated measures ANOVA from post-1 min to post-3 min.

		MEP ratio (mean ± SE)		
Contraction type	Contraction level	Post-1 min	Post-2 min	Post-3 min
Isotonic contraction	10% MVC	74.0 ± 8.1	74.6 ± 9.6	76.6 ± 11.0
	20% MVC	49.9 ± 6.4	66.5 ± 7.8	79.1 ± 10.2
	30% MVC	54.2 ± 11.7	64.8 ± 11.1	86.3 ± 13.8
Isometric contraction	10% MVC	89.7 ± 6.1	107.7 ± 11.9	112.5 ± 9.3
	20% MVC	74.9 ± 10.4	75.0 ± 10.4	87.9 ± 7.9
	30% MVC	71.0 ± 9.7	87.7 ± 13.1	81.2 ± 10.7

**Three-way repeated measures ANOVA**			***F-*value**	***P-*value**

CONTRACTION LEVEL			3.592 (2,22)	*P* < 0.05
CONTRACTION TYPE			5.970 (1,11)	*P* < 0.05
TIME			11.592 (1.240,22)	*P* < 0.01
CONTRACTION LEVEL × TIME			0.550 (4,44)	n.s.
CONTRACTION TYPE × TIME			0.904 (2,22)	n.s.
CONTRACTION LEVEL × CONTRACTION TYPE			0.448 (2,22)	n.s.
CONTRACTION LEVEL × CONTRACTION TYPE × TIME			3.179 (4,44)	*P* < 0.05

**Two-way repeated measures ANOVA**			***F-*value**	***P-*value**

Post-1 min		
CONTRACTION LEVEL			5.509 (2,22)	*P* < 0.05
CONTRACTION TYPE			7.407 (1,11)	*P* < 0.05
CONTRACTION LEVEL × CONTRACTION TYPE			0.177 (2,22)	n.s.
Post-2 min		
CONTRACTION LEVEL			2.751 (2,22)	*P* < 0.1
CONTRACTION TYPE			7.684 (1,11)	*P* < 0.05
CONTRACTION LEVEL × CONTRACTION TYPE			0.503 (2,22)	n.s.
Post-3 min			
CONTRACTION LEVEL			0.976 (2,22)	n.s.
CONTRACTION TYPE			1.863 (1,11)	n.s.
CONTRACTION LEVEL × CONTRACTION TYPE			2.106 (2, 22)	n.s.

*n.s., not significant.*

**Table 2** shows the average amplitudes of the smoothing EMG signals under all conditions. A paired *t*-test showed no statistical difference in the average amplitudes of the smoothing EMG signals in start_60 and end_60 s under all conditions.

**Table 2:** Average amplitudes of smoothing EMG signals (mean ± standard deviation) at start_60 and end_60 s in experiments 1 and 2.

Experiment 1		Start_60 s	End_60 s
Isotonic contraction	10% MVC	11.1 ± 3.7	10.4 ± 4.0
	20% MVC	21.9 ± 6.8	23.6 ± 8.0
	30% MVC	31.4 ± 9.7	33.2 ± 10.6
Isometric contraction	10% MVC	11.1 ± 2.6	10.5 ± 2.2
	20% MVC	20.3 ± 3.1	19.3 ± 3.3
	30% MVC	30.5 ± 2.8	29.2 ± 3.5

**Experiment 2**		**Start_60 s**	**End_60 s**

2-min task		11.1 ± 3.8	10.0 ± 3.9
6-min task		8.5 ± 1.7	8.1 ± 1.9

*Mean ± SD(%).*

### Experiment 2: Effect of Movement Duration

**FIGURE 4:**
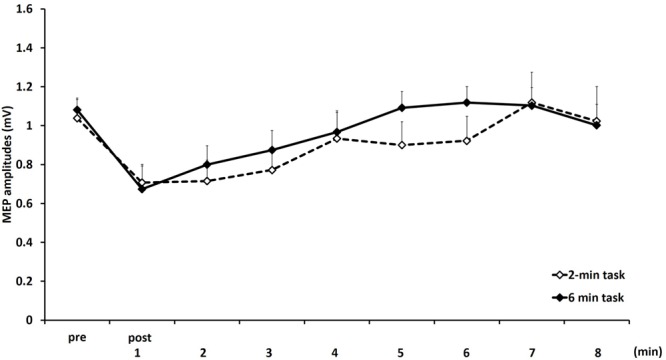
**Means and standard errors of the MEP amplitudes before movement tasks (pre) and 1–8 min after movement tasks at 2- and 6-min task in experiment 2.** Dotted line indicates 2-min task and continuous line indicate 6-min task.

**FIGURE 5:**
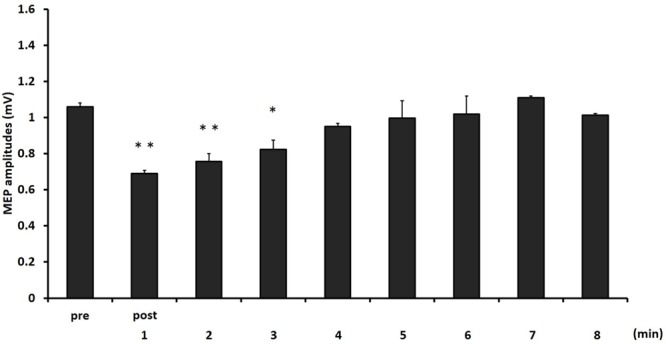
**The comparison of means and standard errors of the MEP amplitudes at different times after movement task in experiment 2 [^∗^*P* < 0.05, ^∗∗^*P* < 0.01 (vs. pre)]**.

**Figure 4** shows the mean MEP amplitudes induced by TMS under 2- and 6-min task. No statistical difference was found in the MEP amplitudes at pre (*P* = 0.471). A two-way repeated measures ANOVA revealed a significant main effect for TIME [*F*_(8,153)_ = 4.284, *P* < 0.01]. However, no statistical difference was found for the main effects of MOVEMENT DURATION [*F*_(1,153)_ = 1.927, *P* = 0.167] and the MOVEMENT DURATION × TIME interaction [*F*_(8,153)_ = 0.384, *P* = 0.928]. *Post hoc* analyses demonstrated that the MEP amplitudes at post-1 min (*P* < 0.01), post-2 min (*P* < 0.01), and post-3 min (*P* < 0.05) were significantly smaller those at pre (**Figure 5**).

A paired *t*-test revealed no significant difference between the average amplitudes of the smoothing EMG signals in start_60 and end_60 s under both conditions and that there is no statistical difference in EMG activity between 2- and 6-min task (*P* = 0.350).

## Discussion

We investigated whether the differences in levels, types, and duration of muscle contraction had an effect on the degree of PED. Our findings showed the degree of PED at post-1 min after 20 and 30% MVC tasks to be greater than that after 10% MVC tasks, and the degree of PED after isotonic contraction tasks to be greater than that after isometric contraction tasks. However, with regard to duration of movement on the 10% MVC task, no difference was found in the degree of PED after 2-min task when compared with 6-min task. These findings showed that the degree of PED immediately after repetitive non-exhaustive movement depended on contraction levels and types, but that the degree of PED after the 10% MVC task was independent of duration of movement.

### PED after Non-exhaustive Movement

No significant difference was found in experiments 1 and 2 in average amplitudes of the smoothing EMG signals at start_60 and at end_60 s. These findings indicate that the movement tasks in this study did not cause muscle fatigue. In addition, the MEP amplitudes significantly decreased after movement tasks in both experiments. Consequently, this study demonstrated that PED was induced after non-exhaustive movement without muscle fatigue. As described in previous studies, PED is induced after non-exhaustive movement without muscle fatigue (Bonato et al., 2002; McDonnell and Ridding, 2006; Avanzino et al., 2011; Teo et al., 2012b; Miyaguchi et al., 2013). Therefore, the findings of the present study showed PED after movement tasks to be consistent with the findings of previous studies.

### Effects in Muscle Contraction Levels and Types

This study demonstrated that the degree of PED at post-1 min after 20 and 30% MVC tasks was greater than that after 10% MVC tasks. van Duinen et al. (2008) compared brain activity during index finger abduction movement tasks in instances of 5, 15, 30, 50, and 70% of maximum force. Their results indicated that the levels of activity in the contralateral sensorimotor cortex area, premotor area, and ipsilateral cerebellum area during movement tasks increased with increasing force levels. Furthermore, Thickbroom et al. (1998) reported that the levels of activity in the sensorimotor cortex area during finger-flexion, movement tasks increased with increasing contraction levels at 5%, 10%, 25%, and 50% MVC. These previous studies indicate that the levels of activity in the motor cortex area during 20 and 30% MVC tasks are greater than those during 10% MVC tasks. In this study, it is considered that the difference in the degree of PED on the difference in muscle contraction levels may be related to the levels of activity in the motor cortex during movement task.

In addition, the present study demonstrated that the degree of PED at post-1 min after isotonic contraction tasks is greater than that after isometric contraction tasks. A number of studies reported that the level of activity in the motor cortex during isotonic contraction is greater than that during isometric contraction (Kasai et al., 1997; Yahagi et al., 2003; Saito et al., 2014). Yahagi et al. (2003) and Saito et al. (2014) used the TMS method to measure MEP amplitudes during isotonic and isometric contraction. Their findings showed that primary motor cortex activity during isotonic contraction is greater than that during isometric contraction, because measured MEP amplitudes during isotonic contraction are larger than those measured during isometric contraction. Moreover, when Yahagi et al. (2003) used the TMS method to study active motor threshold (aMT), they found motor threshold during isotonic contraction to be lower than that during isometric contraction, despite of the identical background EMG activity levels. These previous studies suggest that the level of activity in the motor cortex area during isotonic contraction tasks is larger than that during isometric contraction tasks. The difference in the degree of PED between isotonic and isometric contraction tasks in this study may be associated with the levels of activity in the motor cortex during movement task, as well as the results in muscle contraction levels. In addition, our previous study demonstrated that PED occurred not only after repetitive active movement but also after repetitive passive movement task. Furthermore, it demonstrated that anodal transcranial direct current stimulation (tDCS) over the primary motor area reduced PED after passive movements but not after active movements (Miyaguchi et al., 2013). Area 4, which participates in the movements, has been shown to be activated not only by active movement but also by passive movement (Terumitsu et al., 2009; Onishi et al., 2013). Reddy et al. (2001) have found that area 4 is activated by both active and passive movements and that the activity in area 4 induced by active movement is larger than that induced by passive movement. Based on these studies, the results of our previous study suggested that the degree of PED depends on differences in levels of activity in area 4 and supported the findings of our present studies.

The results of this study suggested that the degree of PED depends on the levels of activity in the motor cortex area during movement tasks. However, some limitations to experiment 1 exist. First, this study cannot precisely clarify whether the isometric contraction tasks produced muscle fatigue. Even though the amounts of EMG signals during isometric contractions were consistent without feedback, the possibility that the increased EMG is canceled out by the decreased contraction level because of fatigue cannot be excluded. However, it was not the case of isotonic contraction because the workload was mostly consist. Therefore, it was considered that it was unlikely to produce fatigue with the isometric contraction with the effort similar with isotonic contraction. Second, the dependencies of PED on the contraction levels and types were observed only at post-1 min. Because of the involvement of multiple factors (3 contraction levels, 2 contraction types, 11 time points) in the analysis, the interpretation of the results of this study leaves room for discussion. We would like to identify the effect of the type and intensity of contraction on PED separately so that we can get deeper insight into the PED mechanism in the next step of our study. Finally, we did not investigate the level of activity in the motor cortex area during movement tasks. We would like to conduct further studies to investigate the level of activity in the motor cortex area during movement tasks.

### Effect in Movement Duration

In experiment 2, we investigated the change in MEP amplitudes under 2- and 6-min task to determine whether the degree of PED was affected by differences in duration of movement on motor tasks. Our findings showed that MEP amplitudes after movement tasks significantly decreased from post-1 min tasks to post-3 min tasks. However, no difference was found in the change in MEP amplitudes between 2- and 6-min task. These findings indicate that the degree of PED during the 10% MVC task is independent of duration of movement. Crupi et al. (2013) compared MEP amplitudes after sequences of opposition movements of the thumb at a frequency of 2 Hz for 5 and 10 min. Their findings suggested that expression of PED depends on the duration of movement because PED was confirmed after the 10-min movement task, but not after the 5-min movement task. In the present study, the movement task consisted of repetitive, index-finger abduction movement at 10% MVC, and MEPs were recorded from FDI. In an earlier study, the movement task consisted of sequences of opposition movements of the thumb to the index, medium, ring, and little fingers, and MEPs were recorded from the FDI (Crupi et al., 2013). Thus, it is possible that the contraction levels of FDI in the present study were larger than those in the study reported by Crupi et al. (2013). For this reason, we suspect that the difference in findings between the present study and the study by Crupi et al. (2013) arose because of the difference in intensity of movement.

## Conclusion

The present study demonstrated that the degree of PED immediately after non-exhaustive repetitive finger movement depends on muscle contraction levels and types. The degree of PED after isotonic contraction tasks was greater than that after isometric contraction tasks, and the degree of PED increased with increasing muscle contraction levels. However, the PED after the 10% MVC task was independent of duration of movement. In brief, our findings suggest that the degree of PED immediately after non-exhaustive repetitive finger movement depends on levels of activity in the motor cortex during movement tasks.

## Author Contributions

HO conceived the study and designed the experiments. SK and SM conducted the experiments. HO, HT, and HK performed interpretation of data. SK and SM performed the statistical analysis. HT, HK and SK wrote and revised the manuscript. HO and SM wrote the manuscript. All authors read and approved the final manuscript.

## Conflict of Interest Statement

The authors declare that the research was conducted in the absence of any commercial or financial relationships that could be construed as a potential conflict of interest.
